# Fanconi Bickel Syndrome: Novel Mutations in *GLUT 2* Gene Causing a Distinguished Form of Renal Tubular Acidosis in Two Unrelated Egyptian Families

**DOI:** 10.1155/2011/754369

**Published:** 2011-07-28

**Authors:** Mohammad Al-Haggar, Osamu Sakamoto, Ali Shaltout, Amany El-Hawary, Yahya Wahba, Dina Abdel-Hadi

**Affiliations:** ^1^Department of Pediatrics, Genetics Unit, Mansoura University Children's Hospital, P.O. 35516, Mansoura, Egypt; ^2^Department of Pediatrics, Tohoku University School of Medicine, Miyagi 980-8575, Japan

## Abstract

*Background*. Fanconi-Bickel syndrome (FBS) is an autosomal recessive disorder caused by defects in facilitative glucose transporter 2 (GLUT2 or SLC2A2) gene mapped on chromosome 3q26.1-26.3, that codes for the glucose transporter protein 2. *Methods*. Two unrelated Egyptian families having suspected cases of FBS were enrolled after taking a written informed consent; both had positive consanguinity, and index cases had evidences of proximal renal tubular defects with hepatomegaly; they were subjected to history taking, signs of rickets as well as anthropometric measurements. Laboratory workup included urinalysis, renal and liver function tests including fasting and postprandial blood sugar; serum calcium, phosphorus, alkaline phosphatase, sodium and potassium, lipid profile, and detailed blood gas. Imaging including bone survey and abdominal ultrasound, and liver biopsy were done to confirm diagnosis. Molecular analysis of the GLUT2 gene was done for DNA samples extracted from peripheral blood leukocyte. All coding sequences, including flanking introns in GLUT2 gene, were amplified using PCR followed by direct sequencing. *Results*. Two new mutations had been detected, one in each family, in exon 3 two bases (GA) were deleted (c.253 254delGA) and in exon 6 in the second family, G-to-C substitution at position-1 of the splicing acceptor site (c.776-1G>C or IVS5-1G>A). *Conclusion*. FBS is a rare disease due to mutation in GLUT2 gene; many mutations were reported, about half were novel mutations; yet none of these mutations is more frequent. A more extensive survey for the most frequent mutations among FBS has to be contemplated to allow for use of molecular screening tests like ARMS.

## 1. Introduction

Fanconi-Bickel syndrome (FBS) is a single-gene disorder (OMIM 227810) caused by defects in the facilitative glucose transporter 2 (GLUT2 or SLC2A2) gene mapped on chromosome 3q26.1-26.3, that codes for the glucose transporter protein 2 expressed in hepatocytes, pancreatic beta cells, enterocytes, and renal tubular cells [1–3]. FBS is a rare inherited disorder of carbohydrate metabolism; it is characterized by the association of huge hepatomegaly due to glycogen accumulation (classified as glycogen storage disease type XI; GSD XI), severe hypophosphatemic rickets and failure to thrive due to proximal renal tubular dysfunction. Proximal renal tubular dysfunction is documented by glucosuria, phosphaturia, generalized aminoaciduria, bicarbonate wasting, and hypophosphatemia; these findings are the characteristic laboratory evidence of the disease. The disorder has been reported from all parts of Europe, Turkey, Israel, Arabian countries of the Near East and North Africa, Japan, and North America. The exact frequency is not known, but the presence of consanguinity in the affected families suggests an autosomal recessive inheritance [1, 2].

In the last decade, many mutations concerning the GLUT2 gene have been described for FBS. In 1997, Santer et al. described the basic defect of this disease on reporting homozygosity for mutations within the GLUT2 gene in four patients [4]. These mutations represented the first detection of a congenital defect within a whole family of membrane proteins, which are the facilitative glucose transporters. Later, Santer et al. reported a total of 109 cases from 88 families worldwide who had been diagnosed as FBS [5]. They reported their results of mutation analysis in 49 patients from 39 families from Turkey, Europe, the Near East, North Africa, and North America. Homozygosity or compound heterozygosity for GLUT2 mutations was found in 49 patients among these cases, and 23 novel mutations of the GLUT2 gene were detected. These mutations were scattered over the whole coding sequence of the GLUT2 gene, and mutations have been found in all exons. None of these mutations was particularly frequent, which makes molecular genetic diagnosis laborious. It is interesting that most of the GLUT2 mutations were private and confined to a single family. Of these patients, 12 were Turkish and all had a different mutation [6]. Since the first report of mutations in the GLUT2 gene [4], more than 30 different mutations have been identified, and most of the reported mutations are private and confined to a single family [6].

There are also FBS patients reported as having no detected mutations in the protein-coding region of the GLUT2 gene [5, 7]. An explanation for this situation can be that at least some of patients carry heterozygous long-range deletions not detectable with the applied PCR-based method [5]. 

Sakamoto et al. [3] performed molecular analysis on three Japanese patients and found four novel mutations: a splice-site mutation (IVS2-2A>G), a nonsense mutation (Q287X), and two missense mutations (L389P and V423E). Şimşek et al. found a novel mutation of the GLUT2 gene in a Turkish patient; two bases were deleted with a homozygous pattern in exon 6 of the GLUT2 gene (c.835_836delGA) [8].

## 2. Case Presentation

### 2.1. Family (1)

A four-year-old male child, second born to consanguineous parents, presented with irritability started at the age of 4 months for which he received antispasmodics with no improvement. Electroencephalogram (EEG) revealed bilateral temporal epileptogenic dysfunction with tendency for secondary generalization. Computed tomography (CT) for brain was normal. Patient received multiple antiepileptics with no improvement. Patient subsequently developed hypotonia for which he was referred to *undergo* electromyogram (EMG) which revealed no evidence of lower motor neuron lesion despite of the delayed sitting till the age of 1.5 years. At age of two years, patient developed abdominal enlargement coincidentally with some urinary symptoms in the form of polyuria and polydipsia that were noticed by mother baby was noticed to be underbuilt at that stage. There were no antenatal problems. After their application to a health facility, he was diagnosed as hypophosphatemic rickets, and treatment with oral vitamin D and phosphate supplementation was commenced. He was transferred to Mansoura University Children's Hospital (MUCH) at the age of 2.5 years for reevaluation and assessment. On physical examination, there were evidences of growth retardation length 75 cm [<5th percentile], weight 9.4 kg [<5th percentile], a “doll-like face,” and clinical manifestations of rickets were also noticed. Abdominal examination revealed distension with hepatomegaly; span of the right lobe of liver was 12 cm in midclavicular line, with firm consistency and rounded border. Rest of clinical examination was unremarkable. Patient was admitted three times to MUCH because of pneumonia. Laboratory assessment was focused on the renal and hepatic profiling to explain the urinary symptoms and hepatomegaly in a racketic child; serum calcium was normal (10 mg/dL, normal: 8–10.2), reduced serum phosphorus (2.2 mg/dL, normal: 2.7–4.5), and markedly elevated serum alkaline phosphatase (995 U/L, normal: 145–420 U/L) levels. Liver and kidney function tests were all normal. Lipid profile was not done. Fasting hypoglycemia (33 mg/dL) and postprandial hyperglycemia (125 mg/dL) were recorded. Arterial blood gas analysis revealed metabolic acidosis (pH, 7.23; bicarbonate, 15 mmol/L) with normal anion gap. Serum sodium (Na+) was 110 mmol/L, and serum potassium (K+) was 2.1 mmol/L. Urinary pH was 6.36 (normal: 4.5–8), with 2+ glucosuria, 1+ proteinuria, and 4+ uric aciduria. Radiologically, bone survey revealed diffuse osteopenia with racketic triad; cupping, fraying and widening of metaphyseal ends of radius and ulna with markedly delayed bone age. Abdominal ultrasound revealed hepatomegaly with both kidneys enlarged. Liver biopsy showed marked glycogen accumulation in hepatocytes; so diagnosis of FBS was presumed, and molecular analysis of the GLUT2 gene was called to confirm the diagnosis. Symptomatic treatment with calcitriol, multivitamins, and Shohl's solution was started, and the mother was advised to feed her baby frequent meals with adequate calories (uncooked corn starch) especially before bedtime. Patient has been followed up to age of 3.5 years. Unfortunately, he expired by at the age of 4 years by severe pneumonia and subsequent respiratory failure.

### 2.2. Family (2)

A 2-year-old male child, first born to consanguineous parents, presented at the age of 7 months by delayed sitting. The patient received active vitamin D and oral calcium with no improvement. Mother noticed polyuria, polydipsia, and abdominal enlargement at the age of 1 year; there were no antenatal problems. He was admitted to MUCH at the age of one year for evaluation of severe growth retardation; physical examination revealed growth retardation (length 64 cm [<5th percentile], weight 5.5 kg [<5th percentile], marasmic face and clinical manifestations of rickets. Marked abdominal distension and hepatomegaly were noted; liver span was 13 cm in the midclavicular line with firm consistency and rounded border. The rest of physical examination was normal. Laboratory examinations revealed reduced serum calcium (7.6 mg/dL, normal: 8–10.2), reduced serum phosphorus (1.6 mg/dL, normal: 2.7–4.5), and markedly elevated serum alkaline phosphatase (3224 U/L, normal: 145–420 U/L) levels. Lipogram was uneventful. Serum lipase enzyme was normal 60 U/L (normal 23–375 U/L). Liver function tests revealed elevated SGPT (263 U/L, normal up to 41 U/L), elevated SGOT (620 U/L, normal up to 37 U/L), and reduced serum albumin (3.4 gm/dL, normal 3.8–5.4 gm/dL). Fasting hypoglycemia (40 mg/dL) and postprandial hyperglycemia (185 mg/dL) were recorded. Arterial blood gas analysis revealed metabolic acidosis (pH, 7.3; bicarbonate, 16 mmol/L) with normal anion gap. Serum Na was 120 mmol/L, and serum K was 2.5 mmol/L. Serum creatinine was normal. Urinary pH was 5 (normal: 4.5–8), with 3+ glucosuria, trace proteinuria, and no uric aciduria. Bone survey revealed diffuse osteopenia, racketic triad with delayed bone age. Abdominal ultrasound revealed hepatomegaly with diffuse fatty infiltration and both kidneys enlarged. Liver biopsy showed massive panacinar macrovesicular steatosis with portal tract expansion by fibrosis. Based on these findings FBS was considered, so molecular genetic testing was arranged for GLUT2 gene. Symptomatic treatment with calcitriol, multivitamins and Shohl's solution was started, and the mother was advised to frequently feed her baby with adequate calories (uncooked corn starch) especially before sleep. At age of 2 years, serum calcium, phosphate, and alkaline phosphatase levels were in normal ranges, yet there was no improvement in growth retardation or other specific features of FBS.

## 3. Patients and Methods

Genomic DNA was extracted from peripheral blood leukocyte samples withdrawn from all available family members (both affected and healthy) and was done using the DNA purification Capture Column Kit (Gentra kit). All coding sequences, including flanking introns in GLUT2 gene, were amplified using polymerase chain reaction (PCR). Direct sequencing of all coding exons including flanking introns of *SLC2A2 *(Glut2 gene) was done for the PCR products using a Big Dye Primer Cycle Sequencing kit and ABI 310 Genetic Analyzer (PE Applied Biosystems, Foster City, CA, USA).

The new mutant forms had been detected in the following DNA stretches: for the first family, on direct sequencing of exon 3 including flanking introns of *SLC2A2 *(Glut2 gene), however; for the second family, on direct sequencing of exon 6 including flanking introns of *SLC2A2 *(Glut2 gene).

## 4. Results

**Figure 1:**
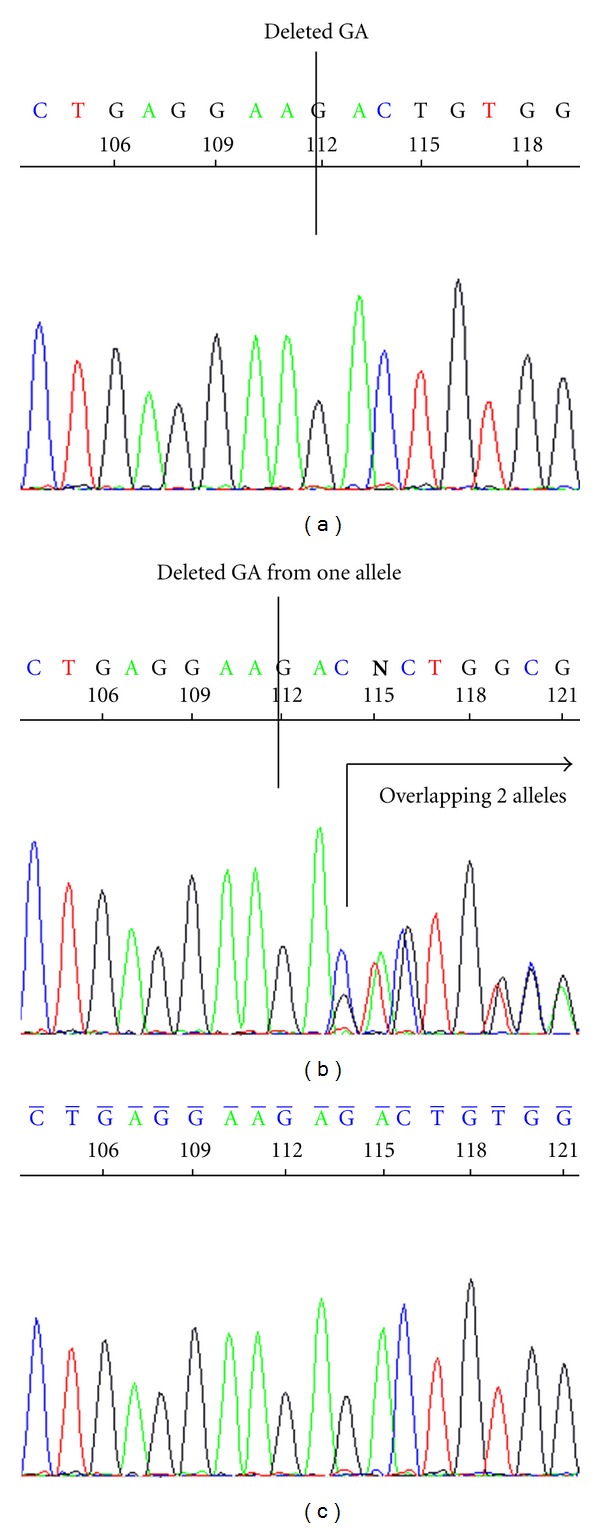
Sequencing exon 3 of *SLC2A2* for family (1) showed the following (a) For patient, two bases (GA) were deleted in a homozygous pattern (c.253_254delGA). (b) Individuals 1101, 1201, 1202, 1203, 1204, 1206, and 1301 were heterozygous, for the above deletion (c.253_254delGA). (c) Individuals 1104, 1205, and 1303 were homozygous normal (mutation c.253_254delGA is not found).

**Figure 2:**
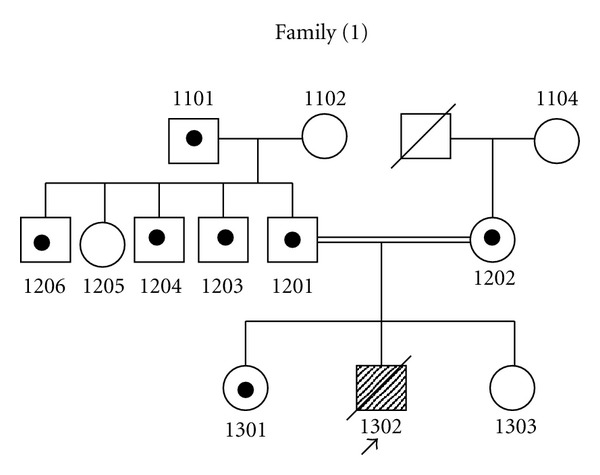
Pedigree of family (1) showing the genotyping of all individuals regarding c.253_254delGA mutation found in the proband (assigned by an arrow); pointed circles and squares are healthy carriers for the mutation while empty circles and squares are healthy individuals tested negative for this mutation.

For family (1), in exon 3 of *SLC2A2*, two bases (GA) were deleted in a homozygous pattern (c.253_254delGA) Figure 1(a). However, individuals 1101, 1201, 1202, 1203, 1204, 1206, and 1301 were heterozygous, for c.253_254delGA (Figure 1(b)), while individuals 1102, 1104, 1205, and 1303 did not show this mutation (Figure 1(c)). Genotyping of family (1) is shown in pedigree (Figure 2).

**Figure 3:**
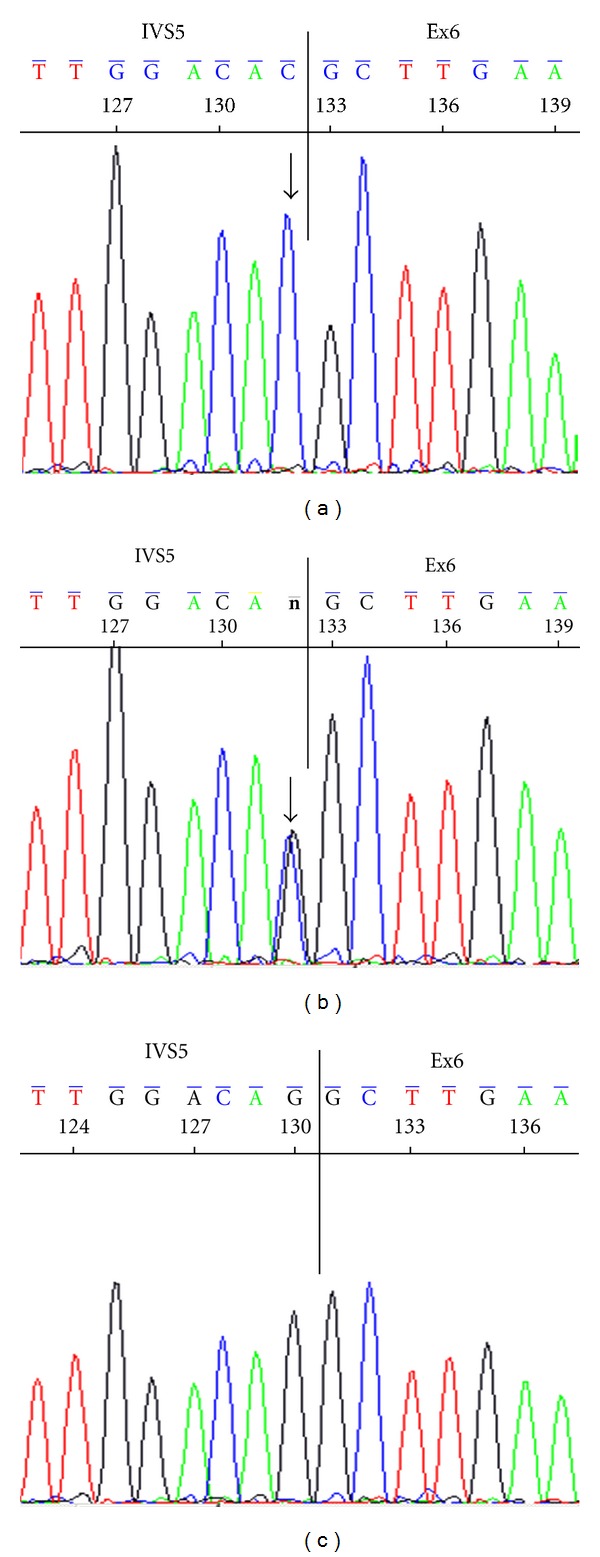
Sequencing of intron5 of *SLC2A2* for family two showed: (a) For patient, there is G-to-C substitution at position-1 of the splicing acceptor site in a homozygous pattern (c.776-1G>C or IVS5-1G>A). (b) Individuals 2102, 2104, 2201, 2202 and 2204 were heterozygous for c.776-1G>C mutation. (c) Individuals 2101, 2103, and 2203 did not show this mutation.

**Figure 4:**
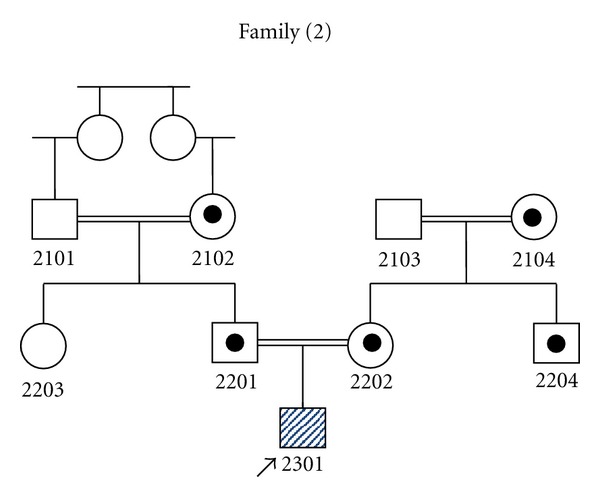
Pedigree of family (2) showing the genotyping of all individuals regarding c.776-1G>C mutation found in the proband (assigned by an arrow), pointed circles and squares are healthy carriers for the mutation while empty circles and squares are healthy individuals tested negative for this mutation.

For family (2), in intron5 of *SLC2A2*, a G-to-C substitution at position-1 of the splicing acceptor site was found in a homozygous pattern (c.776-1G>C or IVS5-1G>A) (Figure 3(a)). However, individuals 2102, 2104, 2201, 2202, and 2204 were heterozygous for c.776-1G>C mutation (Figure 3(b)), while individuals 2101, 2103, and 2203 did not show this mutation (Figure 3(c)). Genotyping of family (2) is shown in pedigree (Figure 4).

## 5. Discussion

FBS is a rare but distinct clinical entity due to mutation in GLUT2 gene; recently many mutations had been described in both patients (homozygous or double heterozygous) and first-degree relatives (sibs and parents); about half of the newly diagnosed cases are due to novel mutation [2, 6]. 

Difficulty in molecular diagnosis of FBS is that none of the reported mutations is particularly more frequent, so no specific mutation type could be diagnosed by a simpler molecular technique like ARMS, for example.

Among more than 10 families with RTA occurring in consanguineous mating, we have screened two families coping with the clinical diagnosis of FBS; specific mutation in each family was discovered adding a new information to literature and confirming the idea that mutant types in this gene could be unlimited. Therefore, this molecular analysis has to be extended to many other families to survey the most frequent mutant forms among such disease in order to make diagnosis in the future families using screening molecular tests.
